# Environmental cues and individuality shape diel and seasonal antelope behaviour in African drylands

**DOI:** 10.1186/s40462-025-00604-y

**Published:** 2025-10-29

**Authors:** Paul Berry, Melanie Dammhahn, Morgan Hauptfleisch, Robert Hering, Niels Blaum

**Affiliations:** 1 Plant Ecology and Nature Conservation, Institute of Biochemistry and Biology, University of Potsdam, Potsdam, Germany; 2 Ongava Research Centre, Ongava Game Reserve, Namibia; 3 Behavioural Biology, Institute for Neuro- and Behavioural Biology (INVB), University of Münster, Münster, Germany; 4 Joint Institute for Individualisation in a Changing Environment (JICE), University of Münster and Bielefeld University, Münster, Germany; 5 Research Directorate, Namibia Nature Foundation, Windhoek, Namibia; 6 Unit for Environmental Sciences and Management, North West University, Potchefstroom, North West Province South Africa; 7 Biodiversity Research Centre, Namibia University of Science and Technology, Windhoek, Namibia; 8 Ecology/Macroecology, Institute of Biochemistry and Biology, University of Potsdam, Potsdam, Germany

**Keywords:** Springbok, Kudu, Eland, Accelerometers, Activity patterns, Semi-arid savanna

## Abstract

Large herbivores play a central role in dryland ecosystems, influencing vegetation dynamics, nutrient cycling, and trophic interactions. While they are adapted to cope with harsh climates, their persistence is increasingly threatened by anthropogenic pressures. However, the behavioural strategies they use to cope with these combined environmental challenges remain understudied. Using multi-year accelerometer data from springbok (*Antidorcas marsupialis*), greater kudu (*Tragelaphus strepsiceros*), and common eland (*Taurotragus oryx*) in northern Namibia, we examined diel and seasonal behaviour in relation to vegetation greenness (NDVI), temperature, lunar phase, and individual differences. While activity was mainly diurnal, nocturnal behaviour was closely linked to the lunar cycle: during moonlit nights, antelope, particularly springbok, increased feeding and walking while reducing rumination and resting. Seasonal patterns tracked plant phenology, with head-up feeding rising sharply during the woody flush at the onset of the green season, while head-down feeding followed grass growth but declined as the season progressed. Seasonal dynamics differed from studies in other regions, suggesting that prolonged dryness and mild winters favour energy conservation over compensatory feeding. Hierarchical partitioning showed that feeding behaviours were environmentally cued, driven by plant phenology and seasonality, whereas walking, rumination, and resting were shaped mainly by individuality. Ambient temperature added little explanatory power, indicating that long-term rhythms are governed more by vegetation cycles and photoperiod than by thermal conditions. Our findings reveal that external cues such as phenology and moonlight synchronise foraging across individuals, while intrinsic factors contribute most to the variation in walking, ruminating and resting, potentially buffering populations against environmental variability. Recognising the combined influence of environmental cues and individual variation is essential for predicting how dryland herbivores will respond to climate and land-use change.

## Introduction

Wildlife in drylands face multiple, interacting pressures which threaten the persistence of populations [1]. Harsh and highly variable climates, characterised by low and unpredictable rainfall and high potential evaporation rates [2], place strong physiological demands on large mammals [3]. At the same time, habitat fragmentation, such as caused by agriculture, settlements and extensive fencing, is disrupting seasonal movements and restricting access to resources [4–7]. In addition, land degradation, for instance through overgrazing and bush encroachment, is diminishing habitat quality and forage availability [8, 9].

While facing these conditions, wildlife plays a central role in savannas and other dryland ecosystems. Large herbivores influence vegetation dynamics, nutrient cycling, and predator–prey interactions [10–13]. Behaviour is a key mechanism by which animals can both respond to and affect their environment, allowing them to adjust rapidly to changing conditions [14] as well as impacting ecosystem structure and function [15]. Understanding these behavioural mechanisms and their limits is therefore essential for effective wildlife conservation [16, 17].

Despite this ecological importance, many aspects of large African herbivores remain understudied [18]. Traditional behavioural studies relied on direct observation, such as those on springbok (*Antidorcas marsupialis*) [19–21], greater kudu (*Tragelaphus strepsiceros*) [22–25], and common eland (*T. oryx*) [26–28], and have provided valuable insights. However, direct observation is resource intensive as well as limited in temporal scope and in its ability to record nocturnal activity [29, 30]. Therefore, important knowledge gaps remain, including the influence of moonlight on nocturnal foraging and predation risk [31], the role of photoperiod in synchronising seasonal rhythms [32], and the extent of individual variation in behavioural strategies (e.g. [33, 34]).

The development of animal-borne accelerometers has greatly advanced the study of behaviour in free-ranging animals by enabling fine-scale data collection over extended periods [35–37]. Accelerometer studies have revealed, for example, how dryland antelope adjust activity patterns in response to heat extremes [38–40] and have uncovered nocturnal behaviours such as biphasic sleep in giraffe [41]. Methodological advances, particularly the application of machine learning to accelerometer data, have further improved behavioural classification [42].

In this study, we build on earlier work [38, 39] by analysing multi-year accelerometer datasets from springbok, greater kudu, and common eland in the arid savanna of northern Namibia. While our previous studies examined short-term behavioural responses to heat, here we consider longer-term patterns across diel and seasonal cycles. Specifically, we assess how seasonal shifts in behavioural time allocation are influenced by time of year, vegetation greenness, ambient temperature, and individual variation. In considering both extrinsic and intrinsic factors, we aim to better understand the extent to which behaviour enables wildlife populations to cope with environmental variability and global change.

## Methods

### Study area

**Fig. 1:**
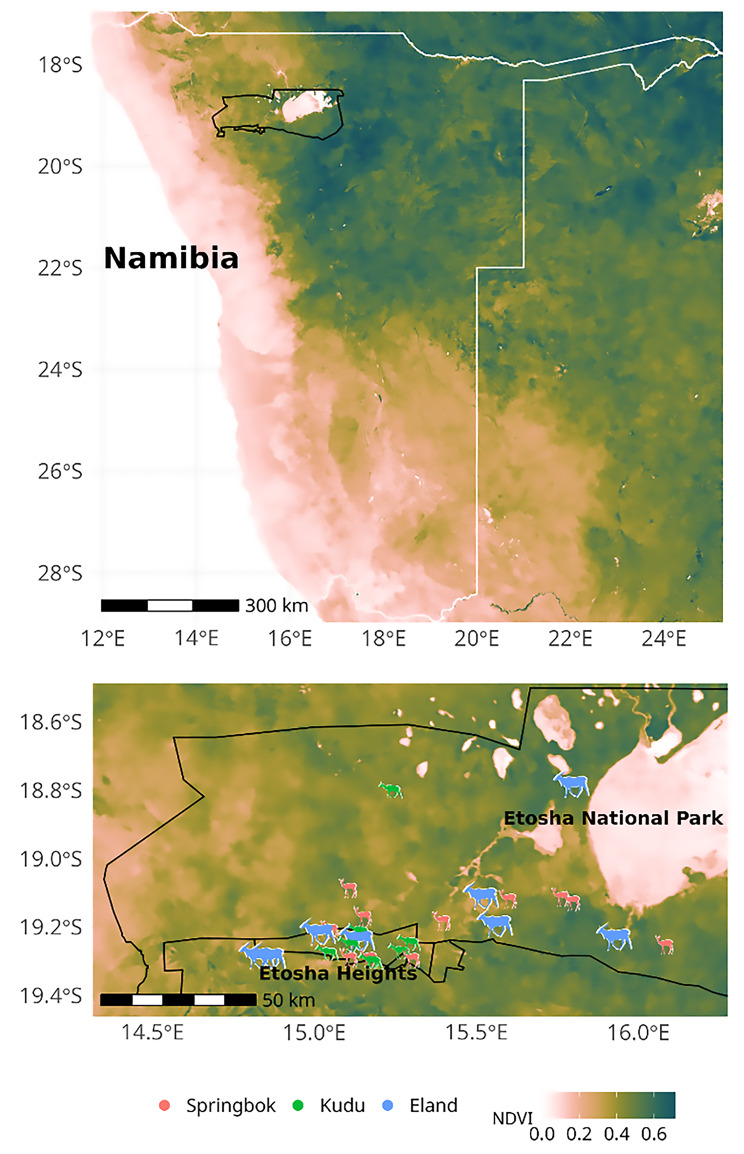
The study took place at Etosha Heights private reserve which borders on the southern boundary of Etosha National Park in Namibia. Icons denote the individual position furthest from their tagging location and colours show species (see legend). Mean NDVI (see legend) during peak green season (February to May 2021) derived vom MODIS data [43] are given at the national scale and within the study region

The study was conducted at the Etosha Heights private reserve (coordinates 19.2°S 15.2°E) which lies along the southern of Etosha National Park, Namibia (Fig. 1). The terrain ranges in elevation from 1,050 to 1,350 metres and is characterised by calcrete plains interspersed with dolomite and limestone hills [44]. The average maximum temperature of the area is 32–34 °C, rainfall is typically limited to the period from October to April and averages 300–350 mm annually while the annual potential evaporation amounts to 2400–2500 mm [45]. The vegetation is classified as a Karstveld tree and shrub savanna [45]. Forage availability in the Etosha region is significantly higher during the wet than the dry season [46]. In addition to the three antelope species studied, the area supports a diverse assemblage of large mammals typical to the Etosha area.

### Study species

Springbok are medium-sized antelope indigenous to southern Africa, with a female body mass of ca. 40 kg. Being mixed feeders [47], springbok consume a variety of grasses, shrubs and ephemerals [19] but tend to browse when woody plant forage is abundant [48]. They are well adapted to arid environments, relying on succulent shrubs to meet their water needs, which allows them to survive without regular access to drinking water [49]. Based on direct observation studies mainly limited to daylight, springbok show peak foraging activity shortly after dawn, around midday, and just before dusk, with these periods being interspersed by two peaks of resting behaviour [20]. Walking is most pronounced during the morning and evening hours [5, 20]. As days shorten, springbok extend their foraging time, while reducing both walking and resting [20].

Greater kudu (hereafter referred to as kudu) are large antelope found in eastern and southern Africa, with a female body mass of approx. 150 kg. Kudu are predominantly browsers [47], with their diet shifting throughout the year depending on availability of palatable vegetation [50]. During the dry season, kudu broaden their diet to include less palatable woody species, and typically increase their feeding time to meet their daily energy needs [23]. The main feeding times are in the early morning and late afternoon, with seasonal differences in time allocations [48]. Temperature can limit their feeding behaviour on hot days [25].

Common eland (hereafter referred to as eland) are distributed across sub-Saharan Africa and are the largest antelope found, with a female body mass of approx. 460 kg [48]. Categorised as mixed feeders [47], they forage on a variety of grazed and browsed species [51]. Despite being well-adapted to arid environments, eland require succulent forage as a source of moisture [48]. Feeding and walking mostly occur in the early morning and late afternoon, with intervening periods of ruminating and resting [26, 27].

### Data collection

Study animals were chemically immobilised by registered veterinarians and fitted with collars equipped with tri-axial accelerometer (ACC) and GPS units (e-obs GmbH, Grünwald, Germany; springbok: Collar 1D, 320 g; eland and kudu: Collar Big 3D, 840 g or Collar Big 4D, 960 g). At the end of the study, animals of which the collar battery still allowed radio tracking were de-collared, while collars of animals that succumbed to predation were retrieved by rangers in the field. All study animals were adults in good condition; most were female, with the exception of one eland male.

The collars were programmed to record ACC data on all three axes at 33 Hz in bursts of 3.3 seconds every 5 minutes. The short burst duration minimised the likelihood of animals transitioning between behaviours within a burst. GPS positions were recorded every 5, 7.5 or 15 minutes, depending on collar battery size; for details see Hering et al. [4]. Data on springbok were recorded from 2019-07-01 to 2022-08-27 on 11 adult females over a total of 5011 individual days (mean number of days per individual: 383, range: 16–1028). Data on kudu were recorded from 2020-07-22 to 2022-10-26 on eight adult females over a total of 5545 individual days (mean number of days per individual: 670, range: 171–827). Data on eland were recorded from 2020-07-21 to 2023-02-19 on eight adult individuals (seven female, one male) over a total number of 4825 individual days (mean number of days per individual: 593, range: 55–944). Permission was granted by the Namibian National Commission on Research, Science and Technology (certificate number RCIV00032018, with authorisation numbers: 20190602, 20190808, and AN202101048) and approved by the Namibian Ministry of Environment, Forestry and Tourism.

### Behaviour classification

To classify behaviour on the basis of ACC data, we used supervised machine learning, which requires labelled data for training and testing. We labelled ACC data with behaviour through direct observation of three springbok, one kudu and one eland, which were additionally collared on Sophienhof farm near the study area. A total of 3952 accelerometer bursts were labelled for springbok as follows: 1188 observations for ID 8316, 1366 observations for ID 8318, and 1398 observations for ID 8320. A total of 2406 bursts were labelled for kudu (ID 8319) and 2876 bursts for eland (ID 7297). The Sophienhof locality was chosen due to the inaccessibility of some parts of the Etosha Heights area, but importantly these animals were also free-ranging and their behaviour did not appear to be affected by the different localities.

The behaviour during ACC recordings was filmed during daylight hours over a period of several weeks. The animals were observed at a distance of 50 to 100 m from a vehicle to which they were habituated, and therefore observer influence was considered negligible. To synchronise the video recordings to the accelerometer data, the NTP-synchronised time displayed on a mobile phone was included in each video and was matched to the GPS time of the accelerometer recording, accounting for the offset between GPS and UTC time.

The video footage was subsequently analysed to label each ACC recording with one of 12 distinct behaviours: browsing, drinking, foraging, grazing, grooming, low-activity, ruminating, running, salt-licking, sleeping, trotting and walking. Foraging, characterised by slow walking with the apparent intent of searching for food, was distinguished from feeding, which involved the intake of food through cropping and chewing while standing. Ruminating was defined as prolonged chewing, often accompanied by occasional regurgitation, but not immediately preceded or followed by feeding. Low-activity behaviours encompassed both standing and lying. A detailed ethogram is provided in [38]. From these 12 behaviours, the most frequently occurring behaviours—feeding (27%), walking (16%), ruminating (27%) and resting (18%)—were further analysed. Feeding was further split into head-up (neck tilt above −30° from the horizontal, interpreted as browsing) and head-down (interpreted as grazing, or browsing near ground level).

The rabc package [52] was used to train a supervised machine learning classifier for each species based on the labelled ACC data. Acceleration data, measured on a scale from 0 to 4095 (corresponding to − 4 G to 4 G), were centred around zero by subtracting 2048 units. To account for variations in collar fit and sensor orientations, the x- and y-axis mean values were compared between the training set and the inference dataset, and axes were reversed if necessary. The magnitude of acceleration in the yz-plane was calculated to account for collar rotation around the neck.

**Fig. 2:**
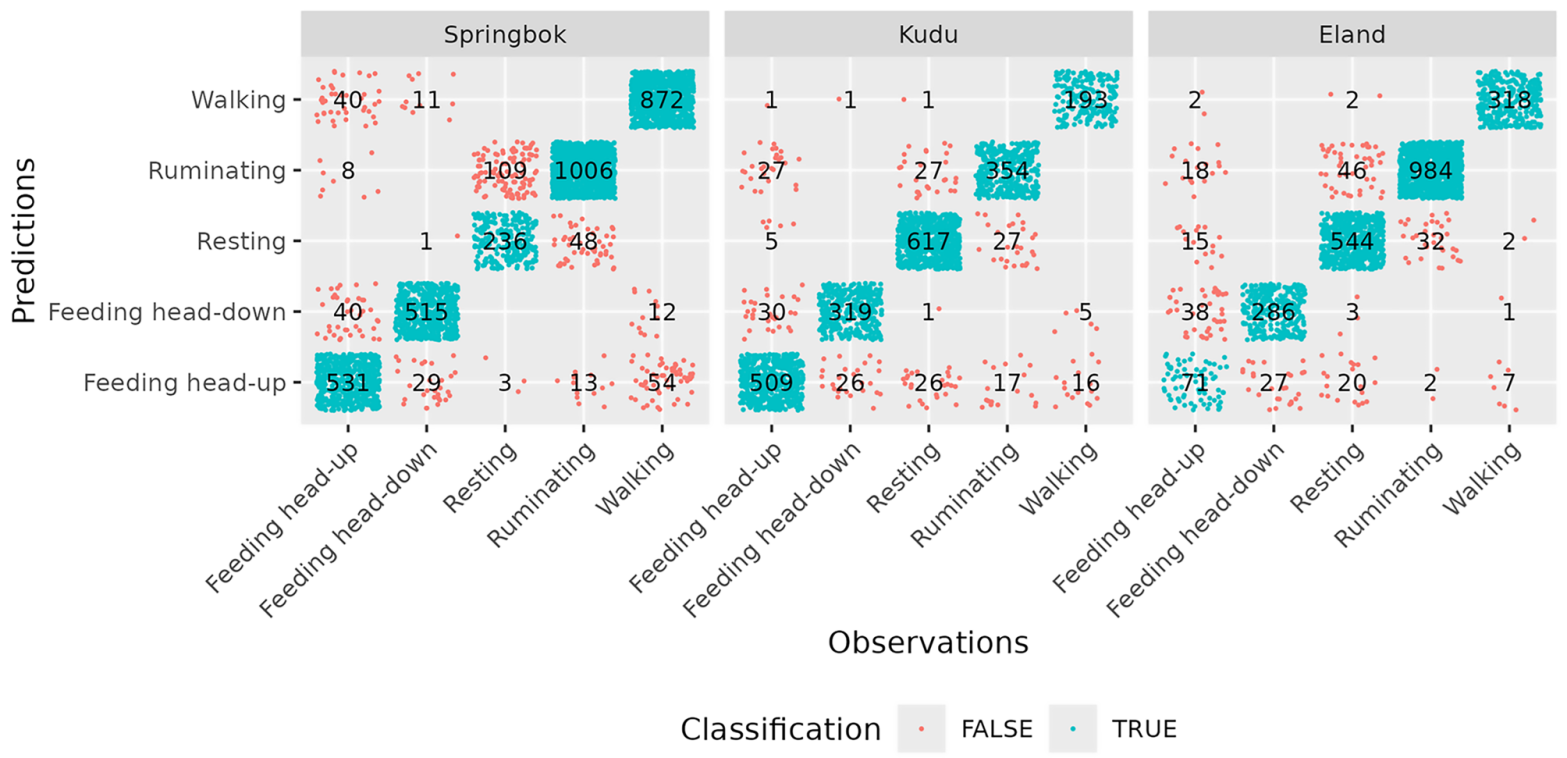
Confusion plots show observed and predicted behaviours in a performance test of the classifier for each species (springbok, kudu, eland). Only the five most frequent behaviours are shown; for less frequent behaviours see Berry et al. [38]

For each 3.3 s continuous recording, several time-domain features (mean, variance, standard deviation, maximum, minimum, range, and overall dynamic body acceleration [ODBA]) and frequency-domain features (main frequency, main amplitude, frequency entropy) were calculated for the x-axis and yz-plane, with ODBA computed across all three axes. The ODBA calculation used a window length of 22 samples (0.67 s). The most predictive features were selected using the rabc package, which removed redundant features based on correlation coefficients (threshold set to 0.9). The selected features for each species and their contribution to classification accuracy were as follows: springbok: x-variance (0.650), x-mean (0.148), yz-entropy (0.032), x-freqmain (0.018), yz-min (0.009); kudu: x-max (0.697), x-freqamp (0.128), x-freqmain (0.029), yz-variance (0.009), yz-entropy (0.007) and eland: yz-variance (0.748), x-min (0.088), x-freqmain (0.020), x-entropy (0.008), x-mean (0.007). Testing of the classifiers indicated that they achieved high accuracy in distinguishing between the main behaviours (Fig. 2).

### Analysis

Acknowledging behavioural changes due to recovery from capture and collaring [53], we disregarded the first 10 days of collected data per individual in our analysis. Also, the last (incomplete) day of accelerometer data recorded for each individual was disregarded to eliminate the effects of de-collaring and predation. The analyses were done using the R language for statistical computing, version 4.4.0 [54].

#### Changes in diel activity patterns across the year

**Fig. 3:**
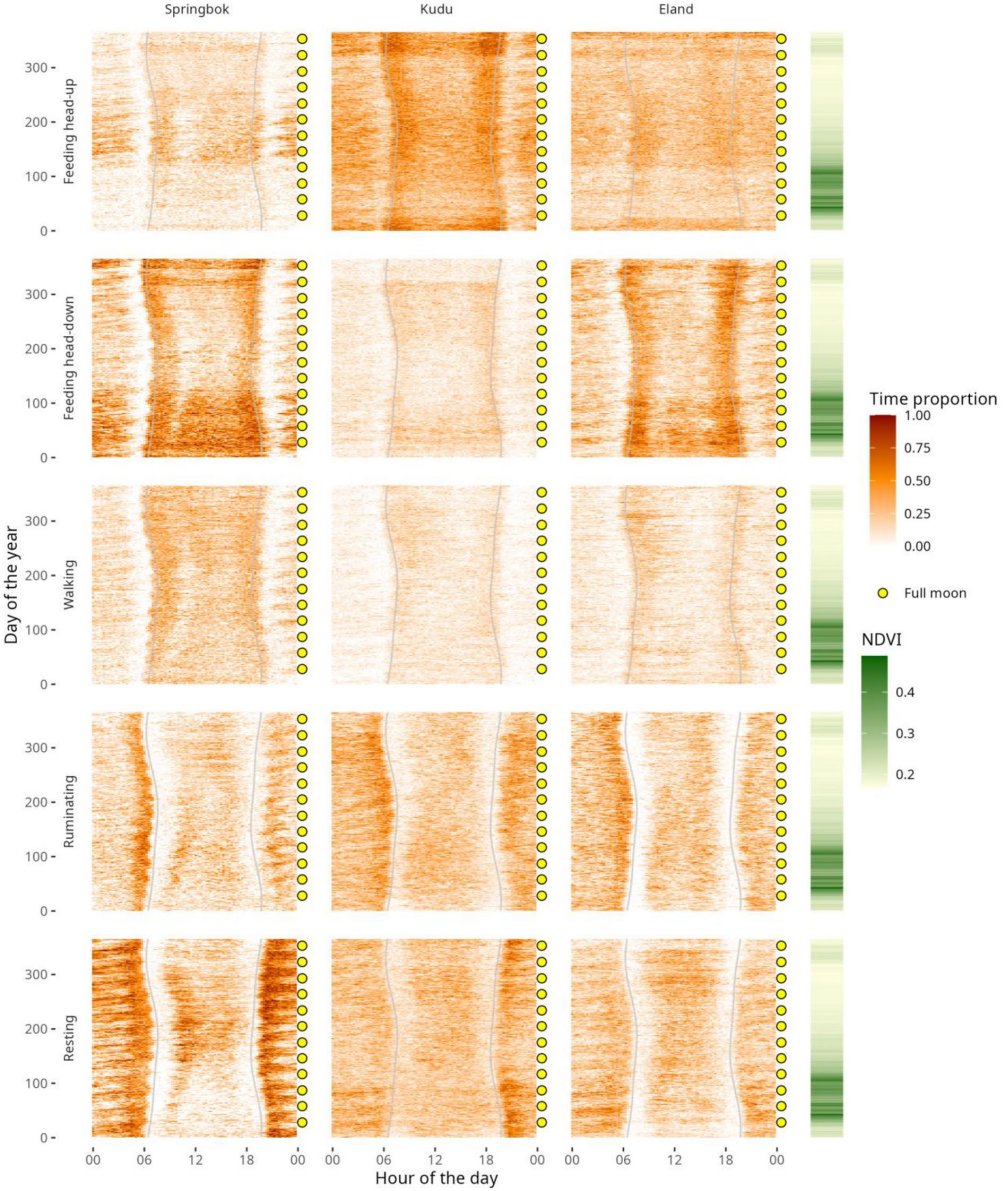
Actograms of springbok, kudu, and eland showing diel and seasonal patterns in the proportion of time allocated to five behaviours (feeding head-up, feeding head-down, walking, ruminating, resting) over the course of one year (2021) in an African dryland savanna. Each panel represents mean values across individuals for each 15-minute interval. Grey shading indicates times of sunrise and sunset. Yellow dots mark nights of full moon, highlighting the synchrony of nocturnal activity with lunar phase. NDVI values to the right of each row show seasonal variation in vegetation greenness

To visualise seasonal changes in diel patterns, we constructed actograms (graphs showing activity patterns over time) for each of the three species based on the mean proportion of time allocated to each of the five behaviours studied. These mean values were calculated across all individuals of a species for each 15-minute-interval of the diel cycle for each day of the year 2021, resulting in 96 (15-minute intervals per 24 h) by 365 values per behaviour per species (Fig. 3). The actograms were annotated to show the synchrony of the diel patterns with lunar phase and of the seasonal patterns with vegetation greenness. Vegetation greenness was quantified by the Normalized Difference Vegetation Index (NDVI) based on MODIS satellite imagery at a 250 m spatial resolution and 16 day temporal resolution. These NDVI data were obtained for each recorded GPS position using the Movebank annotation service [55] and daily average NDVI values were calculated. Hourly displacement was comparable to the spatial resolution of the NDVI data, with means and standard deviations for springbok being 266 ± 373 m, for kudu 170 ± 258 m and for eland 307 ± 462 m. We used NDVI as a proxy of food availability, which has certain limitations: NDVI values can vary due to vegetation characteristics (such as leaf properties, species composition and canopy cover and height) as well as topography and altitude, so that comparing values between adjacent pixels can potentially be misleading [56]. Moreover, we did not differentiate between open and covered areas, although in covered areas the NDVI index reflects the top of the trees, and may not always accurately represent the vegetation encountered by herbivores on the ground [56].

Differences in nocturnal activities between moonlit and dark nights were tested by the Wilcoxon Rank Test. For this, the proportional time allocation to behaviours across all individuals of a species for the hour after midnight (00:00–00:59) were averaged for nights in which fractional moon illumination was more than 0.9 as well as for nights in which it was less than 0.1. The moon illumination for each day was obtained using the suncalc package [57] and ranged fractionally from 0 (New Moon) to 1 (Full Moon).

#### Seasonal variation in time allocation

**Fig. 4:**
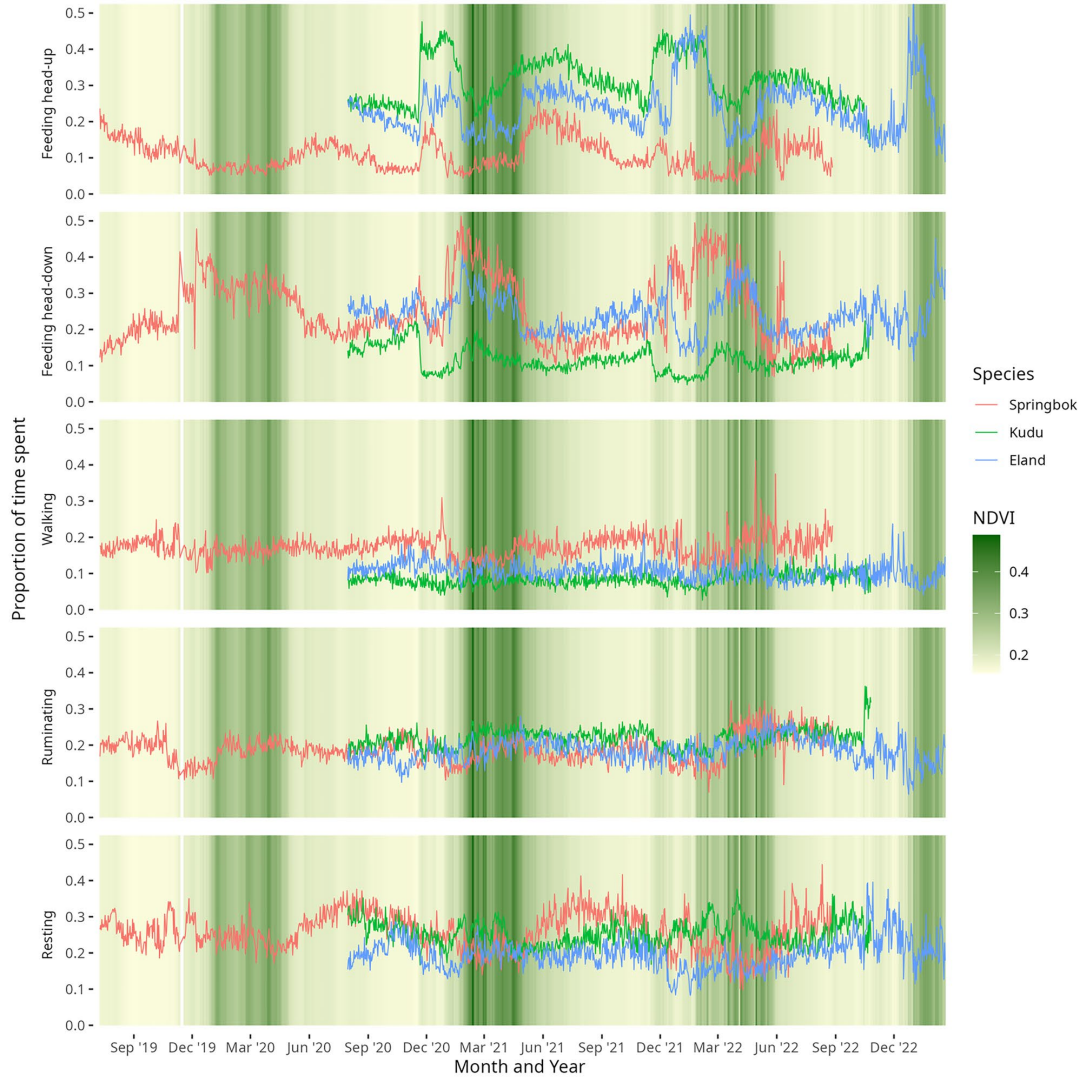
Daily proportion of time allocated to five behaviours—feeding head-up, feeding head-down, walking, ruminating, and resting—by springbok, kudu, and eland from July 2019 to February 2023 in a dryland savanna. Lines represent species means across individuals. Background shading shows daily average NDVI values at the study site, with darker shading indicating higher vegetation greenness

**Fig. 5:**
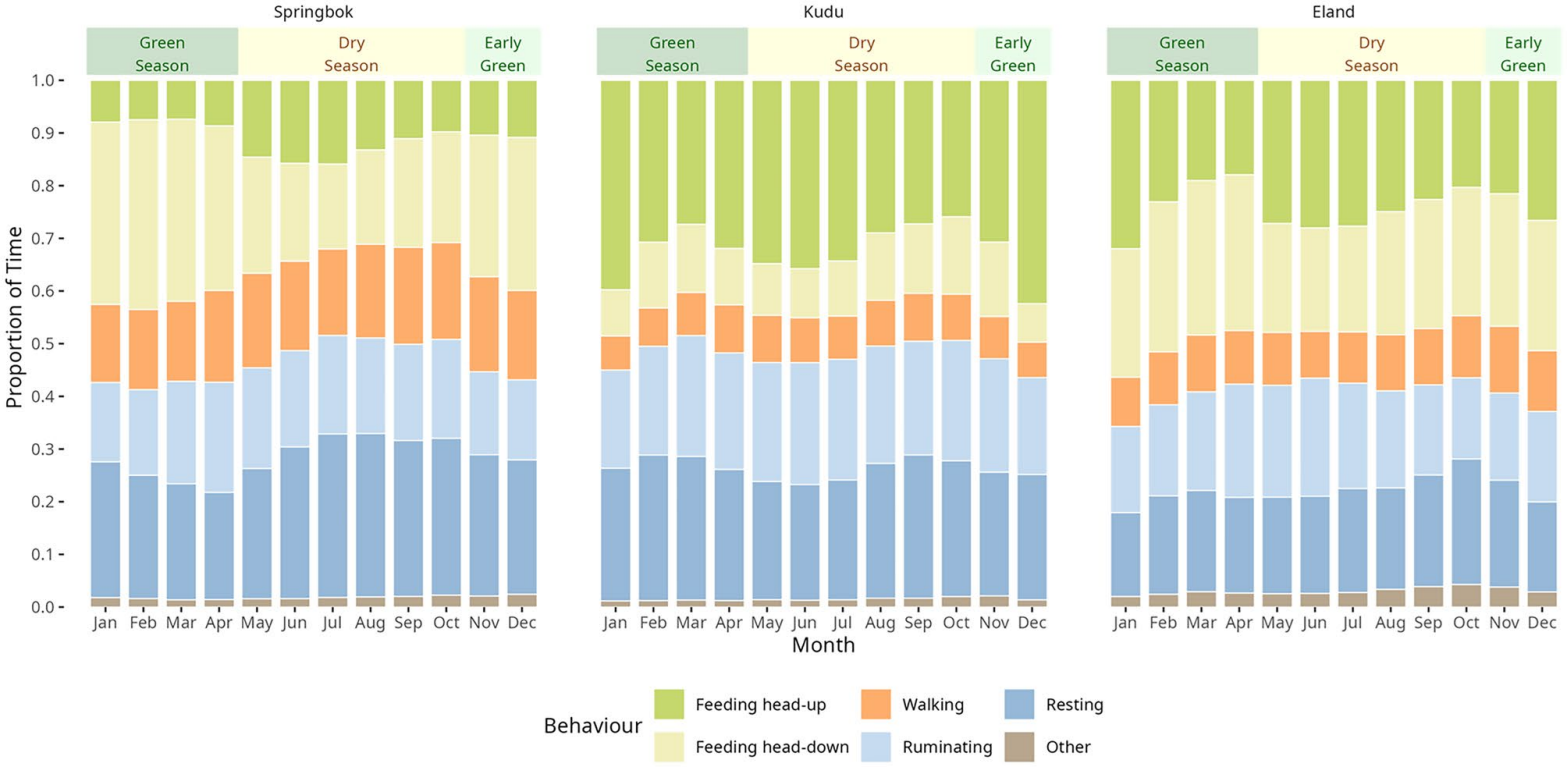
Monthly averages of behavioural time allocation in springbok, kudu, and eland in a dryland savanna. Bars show the proportion of time spent on different behaviours (feeding head-up, feeding head-down, walking, ruminating, resting, and other). Seasonal periods are indicated above each panel: early green season (November–December), green season (January–April), and dry season (May–October)

To visualise inter-day, seasonal, and inter-annual changes in behaviour in relation to vegetation greenness, we plotted the daily proportion of time allocated to the five behaviours, as recorded over a 44 month period from July 2019 to February 2023, for each species (Fig. 4). To further aid the visualisation of seasonal variations, we also plotted monthly averages of time allocated to the five behaviours (Fig. 5), categorising November and December as the early green season, January to April as the green season, and May to October as the dry season.

#### Predictors of seasonal variation

We used the R library mgcv [58] to describe behavioural responses to environmental variables as a Generalised Additive Mixed Model (GAMM) for each of the three species and five behaviours: We related the daily proportion of time (typically based on 288 observations per day and treated as a quasibinomial distribution) spent on the respective behaviour to species (as a factor), month of the year (using cyclic cubic “cc” basis splines), NDVI, and daily mean air temperature. These variables were modelled as smooth terms with the number of basis splines at their default (k = 10). To account for variation among individuals, we included individuals as random intercept effects; model convergence was not possible for random slopes. Air temperature was recorded every five minutes by an on-site weather station. Vegetation greenness, represented by NDVI, was used as a proxy of food availability [59, 60] and daily mean NDVI values were calculated per individual. To model time of year, we used month, rather than day, to capture seasonal trends rather than inter-day variability. The relative importance of each predictor was evaluated using hierarchical partitioning, implemented in the R package gam.hp [61]. Concurvity between average daily temperature and month as well as NDVI was high (<=0.69 and <= 0.72, respectively), so that interpretations on the relative contributions of these variables should be made with caution.

## Results

### Changes in diel activity patterns across the year

The actograms (Fig. 3) reflect the diurnality of all three species, with the active behaviours feeding and walking predominating during the day. Feeding peaked after sunrise and before sunset, the browsing kudu concentrating on head-up feeding and the mixed feeders springbok and eland concentrating on head-down feeding during these times. Of the three species, springbok spent the most time walking, mainly during the day. Ruminating patterns were similar among species, with a peak occurring just before sunrise. Diel resting patterns were most pronounced for springbok, with distinct peaks before sunrise and after sunset. A particularly striking feature of the actograms are the striations reflecting changes in nighttime activity in synchrony with the lunar cycle. Specifically, animals spent more time feeding and walking, and less time ruminating and resting, during moonlit nights compared to dark nights (Table 1). These patterns were most pronounced in springbok. Notably also, the marked seasonal variations in feeding and resting generally affected both daytime as well as nighttime activities. One exception to this is the seasonal variation in resting in springbok, which was characterised by a visible increase during daytime in winter.

**Table 1:** Median proportions of time spent by springbok, kudu, and eland on five main behaviours during moonlit versus dark nights, including Wilcoxon test statistics and p-values

Behaviour	Moonlit	Dark	W	p
**Springbok**				
Feeding Head-Up	0.11	0.07	35247	*<* 0.001
Feeding Head-Down	0.29	0.19	35979	*<* 0.001
Walking	0.17	0.10	40188	*<* 0.001
Ruminating	0.12	0.15	24901	0.033
Resting	0.21	0.35	13837	*<* 0.001
**Kudu**				
Feeding Head-Up	0.34	0.29	18178	*<* 0.001
Feeding Head-Down	0.10	0.08	18062	*<* 0.001
Walking	0.06	0.04	18884	*<* 0.001
Ruminating	0.22	0.27	9766	*<* 0.001
Resting	0.22	0.26	10509	*<* 0.001
**Eland**				
Feeding Head-Up	0.24	0.22	20564	0.061
Feeding Head-Down	0.26	0.17	26956	*<* 0.001
Walking	0.08	0.07	22228	0.001
Ruminating	0.16	0.19	15016	0.001
Resting	0.15	0.23	10569	*<* 0.001

### Seasonal variation in time allocation

Behavioural time allocations, which were averaged by species, varied substantially between days and vegetation greenness varied considerably between years (Fig. 4). The first increase in NDVI during the early green season was associated with a marked increase in head-up feeding in all three species, corresponding to the greening of bushes and shrubs before the onset of the main rainy season and the production of grass. Head-down feeding sharply increased as greening intensified and gradually declined as the green season wore on. Notably, the antelope spent more time feeding during the early and mid green season than in the late dry season (Fig. 5). Walking showed substantial inter-day variation like all behaviours (Fig. 4), but little seasonal variation (Fig. 5). A slight suppression was evident during the green season and a slight elevation during the dry season, most clearly for springbok. Ruminating also showed comparatively little seasonal variation, with a slight suppression during the early part of the green season (Figs. 4 and 5). Resting was elevated during the mid to late dry season in springbok, and during the late dry season in kudu and eland (Fig. 5).

### Predictors of seasonal variation

**Fig. 6:**
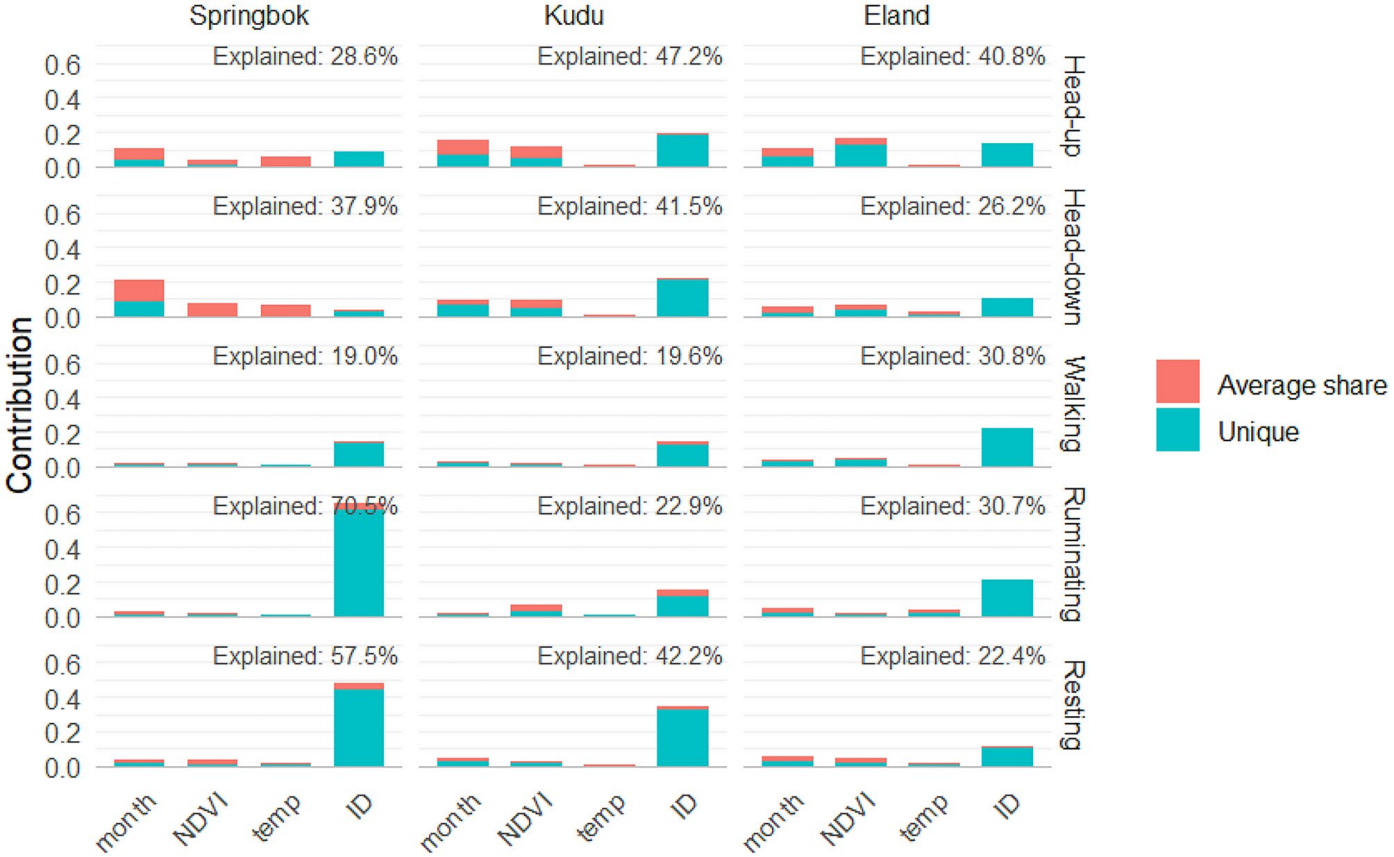
Unique and shared contributions of predictors (month, NDVI, temperature, and individual identity) to behavioural variation in springbok, kudu, and eland. Bars show the contributions of each predictor to the explained deviance of Generalised Additive Models for five behaviours (feeding head-up, feeding head-down, walking, ruminating, and resting). Explained deviance values for each behaviour are indicated in the panels. Results highlight the importance of seasonal and resource-linked drivers (month and NDVI) for feeding behaviours, and the dominant role of inter-individual differences for walking, ruminating, and resting

Hierarchical partitioning showed marked differences in the proportion of deviance explained among behaviours and species, ranging from 0.18 for walking to 0.71 for ruminating, as well as clear variation in the balance between predictors (Fig. 6).

For springbok, in the case of feeding head-up, 0.29 of the deviance was explained, with contributions distributed across month (0.10), temperature (0.06), NDVI (0.04), and individual differences (0.09). For feeding head-down (0.38 of deviance explained), month was the strongest contributor (0.21), followed by NDVI and temperature (both 0.07), with individuality contributing 0.03. Walking (0.19 explained) was mainly accounted for by individuality (0.15), whereas month, temperature, and NDVI each contributed < 0.02. Ruminating achieved the highest explained deviance (0.71), mostly influenced by individuality (0.65). Resting (0.58 explained) was likewise dominated by individuality (0.48), with smaller contributions from month (0.04), NDVI (0.04), and temperature (0.02).

In kudu feeding head-up (0.47 of deviance explained), individuality (0.19) was the strongest contributor, followed by month (0.15) and NDVI (0.12), with temperature contributing negligibly (0.01). Feeding head-down (0.42 explained), was again dominated by individuality (0.22), with NDVI (0.10) and month (0.09) making secondary contributions. Walking (0.20 explained) was again primarily influenced by individuality (0.15), while month, NDVI, and temperature contributed ≤0.03. Ruminating (0.23 explained) was largely explained by individuality (0.15), with a smaller influence from NDVI (0.06). Resting (0.42 explained) showed the same pattern, with individuality (0.34) the dominant factor.

For eland, feeding head-up (0.41 of deviance explained), NDVI (0.17) and individuality (0.13) were the strongest predictors, followed by month (0.10) and temperature (0.01). Feeding head-down (0.26 explained), was driven by individuality (0.10), NDVI (0.07), and month (0.06). Walking (0.31 explained) was again dominated by individuality (0.22). Ruminating (0.31) was largely explained by individuality (0.21), with modest input from month (0.04) and temperature (0.04). Resting (0.22 explained) showed the same trend, with individuality (0.11) the largest contributor.

## Discussion

Large herbivores in drylands face compounding environmental pressures, such as heat, and water and food scarcity [3]. Our analysis of high-resolution, multi-year accelerometer data shows that, while antelope in drylands synchronise their behaviour with environmental cycles, they also show considerable variability between individuals, which may help populations to persist in these highly seasonal arid savannas.

Diel patterns showed peaks in feeding and walking after sunrise and before sunset, and peaks in rumination before dawn, which match observations of earlier studies [20, 25, 27] and which are likely driven by the need to balance foraging with thermoregulation and predation risk [62–64]. Our results extend these long-established patterns by further quantifying nocturnal behaviour. We found that moonlight significantly increased nocturnal feeding, especially in springbok. This result supports the general finding that moonlight facilitates foraging in vision-dependent prey species [31], but contrasts with the notion that moonlight tends to suppress prey activity in open habitats due to predation risk. Possibly, the composition of the predator guild mediates moonlight effects on nocturnal behaviour, with predominantly nocturnal lions generally preferring larger prey than springbok [65].

Seasonal behaviour patterns were closely linked to vegetation dynamics. Head-up feeding increased at the onset of the green season, coinciding with the greening of shrubs and trees, whereas head-down feeding followed grass growth as the rainy season progressed. This may be explained by the Forage Maturation Hypothesis, which predicts that energy intake is maximised when forage is at intermediate phenological stages [66]. The hypothesis is broadly supported across ungulate taxa [67], with smaller-bodied ruminants favouring younger, nutrient-rich forage and larger-bodied species tolerating later, more fibrous material. Our findings align with this: springbok responded strongly to early-season forage while eland showed more generalised feeding patterns. Furthermore, the decreased feeding activity observed during the late dry season may be explained by fibrous, less digestible food passing more slowly through the digestive tract, limiting intake [68, 69]. Importantly however, this reduced feeding contrasts with earlier studies in other regions, where antelope increased feeding during the dry winters to compensate for reduced food availability and quality. For example, springbok in the Karoo rested more during periods of optimal plant growth and increased foraging as pastures deteriorated [20]. In areas with higher rainfall, springbok and eland also increased feeding during the dry winter [28, 70]. These differences may reflect environmental context: our study area experiences a longer dry season with higher average winter temperatures than the Karoo or Highveld. Prolonged dryness may result in more severe food scarcity, while relatively mild winters reduce thermogenic demand, possibly favouring energy conservation rather than compensatory feeding.

The idea of energy conservation as a preferred strategy is further supported by the winter resting behaviour in springbok, which was especially pronounced in the mornings, when animals could absorb warmth from the sun after cold nights. Increased solar absorption during winter has previously been linked to reduced diurnal activity in springbok [71] and absorbing the winter sun through changes in body orientation has been observed as a thermoregulation strategy in a variety of African ungulates [72]. If animals are able to absorb the sun’s rays to help maintain their body temperature, they may need to forage less to cover the costs of homeothermy. In environments where minimum temperatures are relatively mild but food scarcity is prolonged, reliance on behavioural rather than physiological thermoregulation may be the more adaptive strategy, conserving energy reserves to enhance survival and reproductive success [3, 73, 74].

In contrast to previous studies that focused on acute responses to heat [38, 39, 71], temperature explained little additional variance in our models of seasonal activity. While heat events trigger behaviour responses in the short term [40], longer-term patterns in behaviour seem more strongly determined by seasonality and forage availability. This disparity shows the need to specifically consider time scales in the design and the analyses of wildlife behavioural studies related to global change and to differentiate between adaptations to environmental harshness and stress responses [75].

In contrast to feeding, behaviours such as ruminating, resting, and walking were dominated by among-individual differences. This suggests that intrinsic factors, such as physiology, physical condition, or behavioural syndromes, play a greater role than environmental drivers in shaping these behaviours. Individuals may adopt different strategies for energy conservation, which could protect populations against environmental stochasticity [76]. Such individual heterogeneity is increasingly recognised as an important determinant of population resilience [33, 34], but has rarely been quantified in African antelope. Our findings show the importance of accounting for individual variation when predicting population responses to environmental change.

Behavioural patterns, particularly at the seasonal scale, may impact savanna ecosystems, with altered ratios of browsing to grazing potentially influencing the balance between trees and grasses, nutrient cycling, and fire regimes [12]. At the same time, anthropogenic barriers such as fences restrict the ability of ungulates to follow seasonal resources [4, 5, 7]. Because our results show that foraging behaviour is tightly coupled to phenology, fencing and fragmentation are likely to increase nutritional stress by limiting access to forage. Maintaining permeability in dryland landscapes is therefore crucial to protect ungulates against climatic and environmental variability.

In conclusion, our study shows that diel and seasonal behaviours of dryland antelope are formed by both environmental cues and individual differences, with feeding strongly linked to vegetation dynamics and lunar illumination, and other maintenance behaviours largely intrinsically driven. These findings highlight the importance of both extrinsic drivers such as plant phenology, photoperiod, and moonlight, and intrinsic factors such as physiology or behavioural type in understanding the responses of ungulates to environmental change. Comparisons with studies from wetter or cooler regions suggest that prolonged seasonal dryness and relatively mild winters promote energy conservation rather than compensatory feeding. Assessing the relative contributions of environmental synchrony and individual variation and how they vary across timescales is essential for understanding how dryland herbivores respond to climate and land-use change.

## Data Availability

The data supporting the findings of this study have been deposited on the Movebank online platform: https://www.movebank.org, Movebank ID 904829042.

## References

[1] Lewin A, Murali G, Rachmilevitch S, Roll U. Global evaluation of current and future threats to drylands and their vertebrate biodiversity. Nat Ecol Evol. 2024;8:1448–58. 10.1038/s41559-024-02450-4. 38965413 10.1038/s41559-024-02450-4 PMC11310083

[2] Maestre FT, Eldridge DJ, Soliveres S, Kéfi S, Delgado-Baquerizo M, Bowker MA, et al. Structure and functioning of dryland ecosystems in a changing world. Annu Rev Ecol Evol Syst. 2016;47:215–37. 10.1146/annurev-ecolsys-121415-032311. 28239303 10.1146/annurev-ecolsys-121415-032311 PMC5321561

[3] Fuller A, Mitchell D, Maloney SK, Hetem RS, Fonsêca VFC, Meyer LCR, et al. How dryland mammals will respond to climate change: the effects of body size, heat load and a lack of food and water. J Exp Biol. 2021;224:jeb238113. 10.1242/jeb.238113. 33627465 10.1242/jeb.238113

[4] Hering R, Hauptfleisch M, Jago M, Smith T, Kramer-Schadt S, Stiegler J, et al. Don’t stop me now: managed fence gaps could allow migratory ungulates to track dynamic resources and reduce fence related energy loss. Front Ecol Evol. 2022;10:907079. 10.3389/fevo.2022.907079.

[5] Hering R, Hauptfleisch M, Kramer-Schadt S, Stiegler J, Blaum N. Effects of fences and fence gaps on the movement behavior of three southern African antelope species. Front Conserv Sci. 2022;3:959423. 10.3389/fcosc.2022.959423.

[6] Maitima J, Mugatha S, Reid R, Gachimbi L, Majule A, Lyaruu H, et al. The linkages between land use change, land degradation and biodiversity across East Africa. Afr J Environ Sci Technol. 2009;3:310–25.

[7] Stabach JA, Hughey LF, Crego RD, Fleming CH, Hopcraft JGC, Leimgruber P, et al. Increasing anthropogenic disturbance restricts wildebeest movement across East African grazing systems. Front Ecol Evol. 2022;10. 10.3389/fevo.2022.846171.

[8] Kuiper SM, Meadows ME. Sustainability of livestock farming in the communal lands of Southern Namibia. Land Degrad Dev. 2002;13:1–15. 10.1002/ldr.476.

[9] O’Connor TG, Puttick JR, Hoffman MT. Bush encroachment in southern Africa: changes and causes. Afr J Range For Sci. 2014;31:67–88. 10.2989/10220119.2014.939996.

[10] Augustine DJ, McNaughton SJ. Interactive effects of ungulate herbivores, soil fertility, and variable rainfall on ecosystem processes in a semi-arid savanna. Ecosystems. 2006;9:1242–56. 10.1007/s10021-005-0020-y.

[11] Pringle RM, Abraham JO, Anderson TM, Coverdale TC, Davies AB, Dutton CL, et al. Impacts of large herbivores on terrestrial ecosystems. Curr Biol. 2023;33:R584–610. 10.1016/j.cub.2023.04.024. 37279691 10.1016/j.cub.2023.04.024

[12] Staver AC, Abraham JO, Hempson GP, Karp AT, Faith JT. The past, present, and future of herbivore impacts on savanna vegetation. J Ecol. 2021;109:2804–22. 10.1111/1365-2745.13685.

[13] Van der Waal C, Kool A, Meijer SS, Kohi E, Heitkönig IMA, De Boer WF, et al. Large herbivores may alter vegetation structure of semi-arid savannas through soil nutrient mediation. Oecologia. 2011;165:1095–107. 10.1007/s00442-010-1899-3. 21225433 10.1007/s00442-010-1899-3 PMC3057003

[14] Wong BBM, Candolin U. Behavioral responses to changing environments. Behavioral Ecol. 2015;26:665–73. 10.1093/beheco/aru183.

[15] Rahman T, Candolin U. Linking animal behavior to ecosystem change in disturbed environments. Front Ecol Evol. 2022;10. 10.3389/fevo.2022.893453.

[16] Berger-Tal O, Polak T, Oron A, Lubin Y, Kotler BP, Saltz D. Integrating animal behavior and conservation biology: a conceptual framework. Behavioral Ecol. 2011;22:236–39. 10.1093/beheco/arq224.

[17] Bro-Jørgensen J, Franks DW, Meise K. Linking behaviour to dynamics of populations and communities: application of novel approaches in behavioural ecology to conservation. Phil Trans R Soc B. 2019;374:20190008. 10.1098/rstb.2019.0008. 31352890 10.1098/rstb.2019.0008 PMC6710565

[18] Owen-Smith N, Hopcraft G, Morrison T, Chamaillé-Jammes S, Hetem R, Bennitt E, et al. Movement ecology of large herbivores in African savannas: current knowledge and gaps. Mam Rev. 2020;50:252–66. 10.1111/mam.12193.

[19] Bigalke R. Observations on the behaviour and feeding habits of the springbok. 1972, *Antidorcas marsupialis*.

[20] Davies RAG, Skinner JD. Temporal activity patterns of springbok *Antidorcas marsupialis* and merino sheep *Ovis aries* during a karoo drought. Trans R Soc S Afr. 1986;46:133–47. 10.1080/00359198609520114.

[21] Stapelberg H, Van Rooyen MW, Bothma JDP, Van der Linde MJ, Groeneveld HT. Springbok behaviour as affected by environmental conditions in the Kalahari. Koedoe. 2008;50:145–53. 10.4102/koedoe.v50i1.143.

[22] Du Toit JT, Yetman CA. Effects of body size on the diurnal activity budgets of African browsing ruminants. Oecologia. 2005;143:317–25. 10.1007/s00442-004-1789-7. 15605272 10.1007/s00442-004-1789-7

[23] Owen-Smith N. Foraging responses of kudus to seasonal changes in food resources: elasticity in constraints. Ecology. 1994;75:1050–62. 10.2307/1939429.

[24] Owen-Smith N. Assessing the foraging efficiency of a large herbivore, the kudu. S Afr J Wildl Res. 1979;9:102–10. 10.10520/AJA03794369_2784.

[25] Owen-Smith N. How high ambient temperature affects the daily activity and foraging time of a subtropical ungulate, the greater kudu (*Tragelaphus strepsiceros*). J Zool. 1998;246:183–92. 10.1111/j.1469-7998.1998.tb00147.x.

[26] Lewis JG. Game domestication for animal production in Kenya: activity patterns of eland, oryx, buffalo and zebu cattle. J Agric Sci. 1977;89:551–63. 10.1017/S0021859600061323.

[27] Littlejohn A. The feeding behaviour of eland. Br Vet J. 1968;124:335–41. 10.1016/S0007-1935(17)39253-9. 5691504

[28] Underwood R. Social behaviour of the eland (*Taurotragus oryx*) on Loskop Dam Nature reserve (M. Sc. Thesis). 1975. University of Pretoria, Pretoria.

[29] Chadwick FJ, Haydon DT, Husmeier D, Ovaskainen O, Matthiopoulos J. LIES of omission: complex observation processes in ecology. Trends Ecol Evol. 2024;39:368–80. 10.1016/j.tree.2023.10.009. 37949794 10.1016/j.tree.2023.10.009

[30] Smith JE, Pinter-Wollman N. Observing the unwatchable: integrating automated sensing, naturalistic observations and animal social network analysis in the age of big data. J Anim Ecol. 2021;90:62–75. 10.1111/1365-2656.13362. 33020914 10.1111/1365-2656.13362

[31] Prugh LR, Golden CD. Does moonlight increase predation risk? Meta-analysis reveals divergent responses of nocturnal mammals to lunar cycles. J Anim Ecol. 2014;83:504–14. 10.1111/1365-2656.12148. 24102189 10.1111/1365-2656.12148

[32] Hazlerigg DG, Wagner GC. Seasonal photoperiodism in vertebrates: from coincidence to amplitude. Trends Endocrinol Metab. 2006;17:83–91. 10.1016/j.tem.2006.02.004. 16513363 10.1016/j.tem.2006.02.004

[33] Hertel AG, Royauté R, Zedrosser A, Mueller T. Biologging reveals individual variation in behavioural predictability in the wild. J Anim Ecol. 2021;90:723–37. 10.1111/1365-2656.13406. 33301175 10.1111/1365-2656.13406

[34] Réale D, Reader SM, Sol D, McDougall PT, Dingemanse NJ. Integrating animal temperament within ecology and evolution. Biol Rev. 2007;82:291–318. 10.1111/j.1469-185X.2007.00010.x. 17437562 10.1111/j.1469-185X.2007.00010.x

[35] Brown DD, Kays R, Wikelski M, Wilson R, Klimley A. Observing the unwatchable through acceleration logging of animal behavior. Anim Biotelem. 2013;1:20. 10.1186/2050-3385-1-20.

[36] Nathan R, Spiegel O, Fortmann-Roe S, Harel R, Wikelski M, Getz WM. Using tri-axial acceleration data to identify behavioral modes of free-ranging animals: general concepts and tools illustrated for griffon vultures. J Exp Biol. 2012;215:986–96. 10.1242/jeb.058602. 22357592 10.1242/jeb.058602 PMC3284320

[37] Shepard E, Wilson R, Quintana F, Gómez Laich A, Liebsch N, Albareda D, et al. Identification of animal movement patterns using tri-axial accelerometry. Endang Species Res. 2008;10:47–60. 10.3354/esr00084.

[38] Berry P, Dammhahn M, Hauptfleisch M, Hering R, Jansen J, Kraus A, et al. African dryland antelope trade-off behaviours in response to heat extremes. Ecol Evol. 2024;14:e11455. 10.1002/ece3.11455. 38855312 10.1002/ece3.11455 PMC11157150

[39] Berry PE, Dammhahn M, Blaum N. Keeping cool on hot days: activity responses of African antelope to heat extremes. Front Ecol Evol. 2023;11.

[40] Hetem RS, Strauss WM, Fick LG, Maloney SK, Meyer LCR, Shobrak M, et al. Does size matter? Comparison of body temperature and activity of free-living Arabian oryx (*Oryx leucoryx*) and the smaller Arabian sand gazelle (*Gazella subgutturosa marica*) in the Saudi desert. J Comp Physiol B. 2012;182:437–49. 10.1007/s00360-011-0620-0. 22001971 10.1007/s00360-011-0620-0

[41] Burger-Schulz AL, Thiel E, Fennessy J, Fennessy S, Dierkes PW. Accelerometry reveals nocturnal biphasic sleep behavior in wild giraffe. Front Mamm Sci. 2023;2. 10.3389/fmamm.2023.1243883.

[42] Dunford CE, Marks NJ, Wilson RP, Scantlebury DM. Identifying animal behaviours from accelerometers: improving predictive accuracy of machine learning by refining the variables selected, data frequency, and sample duration. Ecol Evol. 2024;14:e11380. 10.1002/ece3.11380. 38756684 10.1002/ece3.11380 PMC11097004

[43] Didan K. MODIS/Terra vegetation indices 16-day L3. In: Global 250m SIN Grid V061. NASA Land Processes Distributed Active Archive Center; 2021. 10.5067/MODIS/MOD13Q1.061.

[44] Nortjé G. A report prepared for Etosha heights game safaris. 2019. University of South Africa.

[45] Atlas of Namibia Team. Atlas of Namibia: its land, water and life. Windhoek: Namibia Nature Foundation; 2022.

[46] Turner WC, Imologhome P, Havarua Z, Kaaya GP, Mfune JKE, Mpofu IDT, et al. Soil ingestion, nutrition and the seasonality of anthrax in herbivores of Etosha National Park. Ecosphere. 2013;4:art13. 10.1890/ES12-00245.1.

[47] Hofmann RR. Evolutionary steps of ecophysiological adaptation and diversification of ruminants: a comparative view of their digestive system. Oecologia. 1989;78:443–57. 10.1007/BF00378733. 28312172 10.1007/BF00378733

[48] Skinner JD, Chimimba CT. The mammals of the southern African subregion. 3rd. Cambridge, New York, Melbourne, Madrid, Cape Town, Singapore, Sao Paulo: Cambridge University Press; 2005. 10.1017/CBO9781107340992.

[49] Nagy KA, Knight MH. Energy, water, and food use by springbok antelope (*Antidorcas marsupialis*) in the kalahari desert. J Educ Chang Mammal. 1994;75:860–72. 10.2307/1382468.

[50] Owen-Smith N, Cooper SM. Nutritional ecology of a browsing ruminant, the kudu (*Tragelaphus strepsiceros*), through the seasonal cycle. J Zool. 1989;219:29–43. 10.1111/j.1469-7998.1989.tb02563.x.

[51] Mtega GA, Nahonyo CL, Temu S, Sangu G, Bukombe J. Habitat use and diet composition of the common eland (Tragelaphus oryx) in Ngorongoro Conservation area, Tanzania. Tanz J Sci. 2023;49:880–90. 10.4314/tjs.v49i4.9.

[52] Yu H, Klaassen M. R package for animal behavior classification from accelerometer data-rabc. Ecol Evol. 2021;11:12364–77. 10.1002/ece3.7937. 34594505 10.1002/ece3.7937 PMC8462134

[53] Stiegler J, Gallagher CA, Hering R, Müller T, Tucker M, Apollonio M, et al. Mammals show faster recovery from capture and tagging in human-disturbed landscapes. Nat Commun. 2024;15:8079. 10.1038/s41467-024-52381-8. 39278967 10.1038/s41467-024-52381-8 PMC11402999

[54] R Core Team. R: A language and environment for statistical computing. R Foundation for Statistical Computing. Vienna: Austria; 2024. https://www.R-project.org/.

[55] Dodge S, Bohrer G, Weinzierl R, Davidson SC, Kays R, Douglas D, et al. The environmental-data automated track annotation (Env-DATA) system: linking animal tracks with environmental data. Mov Ecol. 2013;1:3. 10.1186/2051-3933-1-3. 25709817 10.1186/2051-3933-1-3 PMC4337772

[56] Pettorelli N, Vik JO, Mysterud A, Gaillard J-M, Tucker CJ, Stenseth NC. Using the satellite-derived NDVI to assess ecological responses to environmental change. Trends Ecol Evol. 2005;20:503–10. 10.1016/j.tree.2005.05.011. 16701427 10.1016/j.tree.2005.05.011

[57] Thieurmel B, Elmarhraoui A. Suncalc: compute sun position, sunlight phases, moon position and lunar phase. 2022.

[58] Wood SN. Fast stable restricted maximum likelihood and marginal likelihood estimation of semiparametric generalized linear models. J R Stat Soc Ser B Stat Methodol. 2011;73:3–36. 10.1111/j.1467-9868.2010.00749.x.

[59] Sjöström M, Ardö J, Eklundh L, El-Tahir BA, El-Khidir HAM, Hellström M, et al. Evaluation of satellite based indices for gross primary production estimates in a sparse savanna in the Sudan. Biogeosciences. 2009;6:129–38. 10.5194/bg-6-129-2009.

[60] Wu W, De Pauw E, Helldén U. Assessing woody biomass in African tropical savannahs by multiscale remote sensing. Int J Remote Sens. 2013;34:4525–49. 10.1080/01431161.2013.777487.

[61] Lai J, Tang J, Li T, Zhang A, Mao L. Evaluating the relative importance of predictors in generalized additive models using the *gam.Hp* R package. Plant Divers. 2024;46:542–46. 10.1016/j.pld.2024.06.002. 39280972 10.1016/j.pld.2024.06.002 PMC11390626

[62] Cain JW, Krausman PR, Rosenstock SS, Turner JC. Mechanisms of thermoregulation and water balance in desert ungulates. Wildl Soc Bull. 2006;34:570–81. 10.2193/0091-7648(2006)34%255B570:MOTAWB%255D2.0.CO;2.

[63] Owen-Smith N, Goodall V. Coping with savanna seasonality: comparative daily activity patterns of African ungulates as revealed by GPS telemetry: comparative daily activity patterns of African ungulates. J Zool. 2014;293:181–91. 10.1111/jzo.12132.

[64] Tambling CJ, Minnie L, Meyer J, Freeman EW, Santymire RM, Adendorff J, et al. Temporal shifts in activity of prey following large predator reintroductions. Behav Ecol Sociobiol. 2015;69:1153–61. 10.1007/s00265-015-1929-6.

[65] Hayward M. Prey preferences of the lion (Panthera leo). J Zool. 2006. 10.1017/S0952836905007508.

[66] Fryxell JM. Forage quality and aggregation by large herbivores. Am NaturaList. 1991;138:478–98. 10.1086/285227.

[67] Esmaeili S, Jesmer BR, Albeke SE, Aikens EO, Schoenecker KA, King SRB, et al. Body size and digestive system shape resource selection by ungulates: a cross-taxa test of the forage maturation hypothesis. Ecol Lett. 2021;24:2178–91. 10.1111/ele.13848. 34311513 10.1111/ele.13848

[68] Blaxter KL, Wainman FW, Wilson RS. The regulation of food intake by sheep. Anim Sci. 1961;3:51–61. 10.1017/S0003356100033766.

[69] Hofmann RR, Knight MH, Skinner JD. On structural characteristics and morphophysiological adaptation of the springbok (*Antidorcas marsupialis*) digestive system. Trans R Soc S Afr. 1995;50:125–42. 10.1080/00359199509520344.

[70] Novellie PA. Comparison between the foraging stragies of blesbok and springbok on the Transvaal highveld. S Afr J Wildl Res. 1978;8:137–44.

[71] Hetem RS, de Witt BA, Fick LG, Fuller A, Kerley GIH, Meyer LCR, et al. Body temperature, thermoregulatory behaviour and pelt characteristics of three colour morphs of springbok (*Antidorcas marsupialis*). Comp Biochem Physiol A Mol Integr Physiol. 2009;152:379–88. 10.1016/j.cbpa.2008.11.011. 19056508 10.1016/j.cbpa.2008.11.011

[72] Hetem RS, Maartin Strauss W, Heusinkveld BG, De Bie S, Prins HHT, Van Wieren SE. Energy advantages of orientation to solar radiation in three African ruminants. J Therm Biol. 2011;36:452–60. 10.1016/j.jtherbio.2011.07.012.

[73] van Beest FM, Milner JM. Behavioural responses to thermal conditions affect seasonal mass change in a heat-sensitive northern ungulate. PLoS One. 2013;8:e65972. 10.1371/journal.pone.0065972 23776584 10.1371/journal.pone.0065972 PMC3679019

[74] Blank DA. Using microclimate of arid landscape as a resource in goitered gazelle comfort behavior. J Arid Environ. 2020;180:104201. 10.1016/j.jaridenv.2020.104201.

[75] Schradin C, Makuya L, Pillay N, Rimbach R. Harshness is not stress. Trends Ecol Evol. 2023;38(3):224–27. 36641304 10.1016/j.tree.2022.12.005

[76] Dammhahn M, Landry-Cuerrier M, Réale D, Garant D, Humphries MM. Individual variation in energy-saving heterothermy affects survival and reproductive success. Funct Ecol. 2017;31(4):866–75. 10.1111/1365-2435.12797.

